# Different Multimorbidity Measures Result in Varying Estimated Levels of Physical Quality of Life in Individuals with Multimorbidity: A Cross-Sectional Study in the General Population

**DOI:** 10.1155/2016/7845438

**Published:** 2016-03-16

**Authors:** Aline Ramond-Roquin, Jeannie Haggerty, Mireille Lambert, Jose Almirall, Martin Fortin

**Affiliations:** ^1^Département de Médecine de Famille et de Médecine d'Urgence, Université de Sherbrooke, 3001 12e Avenue Nord, Sherbrooke, Québec, Canada J1H 5H3; ^2^Centre Intégré Universitaire de Santé et de Services Sociaux du Saguenay-Lac-Saint-Jean, 305 Saint-Vallier, Chicoutimi, Canada; ^3^Département de Médecine Générale, Université d'Angers, L'Université Nantes Angers Le Mans, 1 rue Haute de Reculée, 49045 Angers Cedex 01, France; ^4^Laboratoire d'Ergonomie et d'Épidémiologie en Santé au Travail, Université d'Angers, L'Université Nantes Angers Le Mans, 1 rue Haute de Reculée, 49045 Angers Cedex 01, France; ^5^Faculty of Medicine, McGill University, Montreal, Canada

## Abstract

*Introduction*. Multimorbidity adversely affects health-related quality of life. Methodological factors may impact the magnitude of this relationship.* Objective*. To evaluate how physical health-related quality of life varies in individuals with multimorbidity depending on the length of the list of candidate conditions considered.* Methods*. Secondary analysis from PRECISE, a cohort study of the general adult population of Quebec, Canada. Multimorbidity was measured using the 21-chronic condition list from the Disease Burden Morbidity Assessment, and physical health-related quality of life was measured using the physical component summary (PCS) of SF-12v2. The PCS was calculated, (a) using 2 or more conditions from the 21-condition list (MM2+, 21) and then from a reduced 6-condition list (MM2+, 6) and (b) using three or more conditions from each list (MM3+, 21, and MM3+, 6).* Results*. The analysis included 1,710 individuals (mean age 51.3, 40.5% men). Multimorbidity prevalence ranged from 63.8% (MM2+, 21 conditions) to 3.8% (MM3+, 6 conditions). The mean [95% CI] PCS dropped from 45.7 [CI: 45.0–46.3] (MM2+, 21) to 40.2 [CI: 38.7–41.8] (MM2+, 6) and from 44.2 [CI: 43.4–44.9] (MM3+, 21) to 34.8 [CI: 31.9–37.6] (MM3+, 6).* Conclusion*. The length of the list of candidate conditions considered has a great impact on the estimations of physical health-related quality of life.

## 1. Introduction

Prevalence of multimorbidity, which refers to the cooccurrence of multiple chronic conditions in the same individual, has increased over the last decades in the general population [1–3]. Because of its association with multiple negative consequences at the individual, healthcare systems, and societal levels, multimorbidity is now acknowledged by some as a research priority [4–6]. Outcomes associated with multimorbidity still need to be explored.

Health-related quality of life, which is a multidimensional concept that refers to physical, psychological, and social domains of health, is adversely affected by the presence of multimorbidity [7–9]. The increasing number of concurrent chronic conditions has been found to be strongly associated with lower scores of health-related quality of life [10–15]. This association seems to be exacerbated in younger people and in the most deprived populations and perhaps to a lesser extent in women [10, 11, 16, 17]. The physical component of health-related quality of life seems to be more affected by multimorbidity than the mental component [18, 19].

Studies aiming to quantify the impact of multimorbidity on the quality of life show wide heterogeneity in terms of the intensity of this association [18]. It has been suggested that the lack of a uniform way to measure multimorbidity may explain a significant part of this variability. However, these studies also presented other important methodological differences (population studied, measure of quality of life, etc.) which prevented the evaluation of the own impact of multimorbidity measure on the heterogeneity observed.

Multimorbidity can be measured using either simple count of conditions or weighted measures which take into account the severity of each existing condition [20]. The use of weighted measures of multimorbidity seems to reveal a stronger association with health-related quality of life, probably because higher scores do not necessarily mean higher number of chronic conditions [21]. However, most operational definitions of multimorbidity in the literature have been based on a simple count of conditions. In such cases, a minimal number of conditions are required to be considered as “multimorbid,” often two or more conditions (MM2+) or three or more conditions (MM3+) in a single individual [22].

Independent of whether a weighted measure or a simple count is used, an important aspect of its measurement is the list of conditions screened as present or not in a given individual. This methodological aspect applies to every study on multimorbidity. Many different lists of potential conditions have been proposed, with a median number of 14 conditions [20], with some being as short as six conditions [9, 19] and others as long as 40 [17, 23]. It is known that differences in the list of conditions considered to measure multimorbidity have a considerable influence on estimates of the prevalence of multimorbidity [24]. However, the influence of the list of conditions on the estimated level of the physical component of health-related quality of life in individuals with multimorbidity has yet to be investigated.

## 2. Objectives

This study aimed to evaluate how estimates of physical health-related quality of life vary in individuals with multimorbidity depending on the length of the list of candidate conditions, using different operational definitions of multimorbidity.

## 3. Methods

### 3.1. Study Design and Setting

This cross-sectional study builds on a secondary analysis of data collected for a larger project, the Program of Research on the Evolution of a Cohort Investigating Health System Effects (PRECISE) [25]. This project aimed to examine the effects of the transformation of primary healthcare services on a population's health. The PRECISE study was conducted in four local healthcare networks in Quebec, Canada, located in metropolitan, urban, rural, and remote settings. Details of the method and sampling strategies used are described in the study protocol reported elsewhere [25].

### 3.2. Population Recruitment

The present study included a randomly selected sample recruited from March to April 2010 in the general population by random digital dialing. Once contact was made, staff selected the eligible adult in the household with the most recent birthday to ensure random selection. Participants had to be community-dwelling adults, aged between 25 and 75 years, without major cognitive impairment, able to respond to written and oral questions in English or French, and to reside in one of the four networks identified.

### 3.3. Data Collection

At recruitment, participants reported on sociodemographic information: age, gender, household income, education level, perceived financial situation, house ownership, presence or absence of medical insurance, and possession of a retirement plan. We produced a data-driven classification of socioeconomic status based on the last five variables and classified individuals into four clusters: elite group, middle-high, middle-low, and low. This aimed to capture the multidimensional nature of socioeconomic status into a single variable. Definitional criteria for the four socioeconomic clusters are described elsewhere [26].

Two weeks after recruitment, participants completed a self-administered questionnaire (paper or online) or a questionnaire administered by telephone that included sections to measure (1) multimorbidity and (2) physical health-related quality of life.

*Measurement of Multimorbidity*. The instrument comprised a list of 21 chronic conditions adapted from the Disease Burden Morbidity Assessment (DBMA). It has been validated to measure multimorbidity, including validation in a population from Quebec, with a good predictive value for health-related quality of life [8, 27, 28]. To determine the presence of a condition, for 20 out of 21 conditions, the instruction to the participants was as follows: “*Please, indicate if you have been told by a health professional that you have any of the following illnesses*.” For the 21st condition, “overweight,” the participant was invited to self-report his or her height and weight, from which we calculated the Body Mass Index (BMI). We considered the presence of overweight when the BMI was higher than 24.9 Kg/m^2^ [29]. The number of conditions for each individual was first summed up based on the full list of 21 conditions (shown in the results section). We then summed up the number of conditions based on a reduced list of six conditions (out of 21) to correspond to a list previously used in the literature to study health-related quality of life and multimorbidity [9, 19]. Any missing value was considered as an absent condition. We also applied successively two operational definitions of multimorbidity, MM2+ then MM3+. Therefore, multimorbidity was successively measured in four different ways in this study: MM2+ (21); MM2+ (6); MM3+ (21); and MM3+ (6). Given that the list of six conditions was a sublist of the 21 conditions, it follows that, for each of the operational definitions of multimorbidity, the individuals considered as multimorbid according to the 6-condition list constituted a subsample of those considered as multimorbid according to the 21-condition list.

*Measurement of Physical Health-Related Quality of Life*. The physical component of health-related quality of life was measured using the SF-12, version 2 [30, 31], a short form version of SF-36 [32], a generic instrument validated in a Canadian population [33]. The physical component summary is calculated from weighted scores of both the mental and the physical dimensions of the SF-12v2. The physical component summary ranges from 0 to 100, with a population-normed mean of 50 and a standard deviation of 10, where a 5-point difference is considered clinically significant [34]. Lower scores refer to lower physical quality of life.

### 3.4. Data Analysis

We first studied the sociodemographic characteristics and the number of chronic conditions in the whole population, then in individuals with multimorbidity, using successively each of the four multimorbidity measures. We then looked at an assumption underlying the main analyses; namely, whether there was a statistically significant association between multimorbidity and physical health-related quality of life, adjusted for sociodemographic covariates, for each of the four multimorbidity measures.

To evaluate how estimates of physical health-related quality of life vary with the length of the list of candidate conditions considered, we estimated the average level (mean values and 95% confidence intervals) of the physical component of health-related quality of life in individuals with multimorbidity and highlighted the resulting variation using the 21-condition list or the 6-condition list. We conducted these analyses successively using each of the two operational definitions of multimorbidity. Finally, we repeated all these analyses stratifying by age, gender, and socioeconomic status, in order to determine if the variations observed were consistent within each of these subgroups of individuals. For the stratified analysis, age was considered in three groups (25–44 years old, 45–64 years old, and 65–75 years old), based on previous literature on multimorbidity [35].

Categorical variables were described with absolute numbers and percentages. Quantitative variables were described using means and standard deviations (SD). Confidence intervals (95% CI) around estimated means of physical health-related quality of life were calculated using standard errors of the means, with appropriate statistics in the case of small samples. Analysis of covariance (ANCOVA) was used to characterize the association between multimorbidity and physical health-related quality of life, with modelling physical health-related quality of life according to multimorbidity status and sociodemographic covariates. All analyses were done using the SPSS version 20 software.

The study was approved by the ethics committee of the Centre de Santé et de Services Sociaux de Chicoutimi, as well as the research ethics committee of Hôpital Charles Lemoyne, QC, Canada.

## 4. Results

A total of 1,710 individuals participated in the PRECISE study and their data were included in these analyses. Among the study population, mean (SD) age was 51.3 (SD 12.5) years and there were 693 (40.5%) men. The main sociodemographic characteristics of the participants are presented in Table 1.

Regarding chronic conditions, absence of overweight was imputed to 57 individuals due to missing values. For every other condition, there was either 0 or 1 missing value. Prevalence of each individual chronic condition in the study population is shown in Table 2. Considering the 21-condition list, 272 individuals (15.9%) reported no chronic condition, and the mean number of chronic conditions was 2.9 (SD 2.4). Alternatively, when the 6-condition list was considered, 1,016 individuals (59.4%) reported no chronic condition and the mean number of chronic conditions was 0.6 (SD 0.8).

As expected, a clinically and statistically significant association between multimorbidity status and physical health-related quality of life was observed for each of the four multimorbidity measures, adjusting for age, gender, and socioeconomic status. Depending on the multimorbidity measures, adjusted regression parameters associated with multimorbidity ranged from 8.57 to 10.92 (*p* < 0.001 for each parameter).

**Figure 1:**
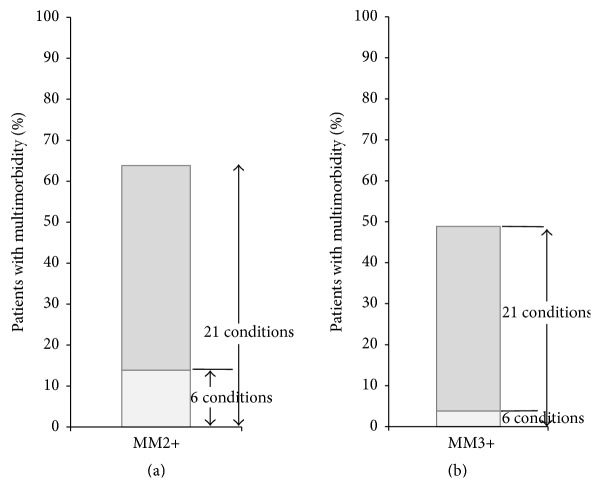
Prevalence of multimorbidity, depending on the multimorbidity measure. Operational definitions of multimorbidity: MM2+: having two or more chronic conditions; MM3+: having three or more chronic conditions.

**Figure 2:**
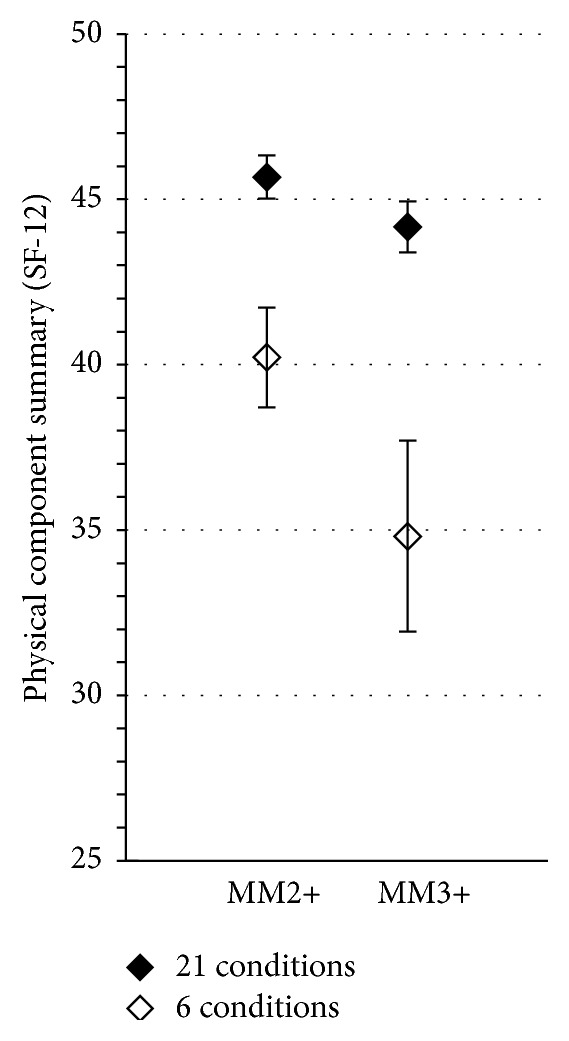
Estimates of the average level (mean values and 95% confidence intervals) of the physical component of health-related quality of life in individuals with multimorbidity, depending on the multimorbidity measure. Operational definitions of multimorbidity: MM2+: having two or more chronic conditions; MM3+: having three or more chronic conditions.

Prevalence of multimorbidity as well as level of physical health-related quality of life in those considered as multimorbid largely varied depending on the multimorbidity measure used (Figures 1 and 2). Using the MM2+ definition, individuals with multimorbidity defined by the 21-condition list (*n* = 1091, 63.8% of the total population) had a mean physical component summary (95% CI) of 45.7 (CI: 45.0–46.3), while the group defined by the 6-condition list (*n* = 237, 13.8% of the total population) scored 40.2 (CI: 38.7–41.7) on average. Using the MM3+ definition, individuals with multimorbidity defined by the 21-condition list (*n* = 836, 48.9% of the total population) had a mean physical component summary (95% CI) of 44.2 (CI: 43.4–44.9), while the group defined by the 6-condition list (*n* = 66, 3.8% of the total population) scored 34.8 (CI: 31.9–37.6) on average.

Regarding sociodemographic variables, using a reduced list of conditions led to the selection of a subgroup of older and more deprived individuals, with a higher proportion of men (Table 3). This was true for both operational definitions of multimorbidity (MM2+ and MM3+).

**Figure 3:**
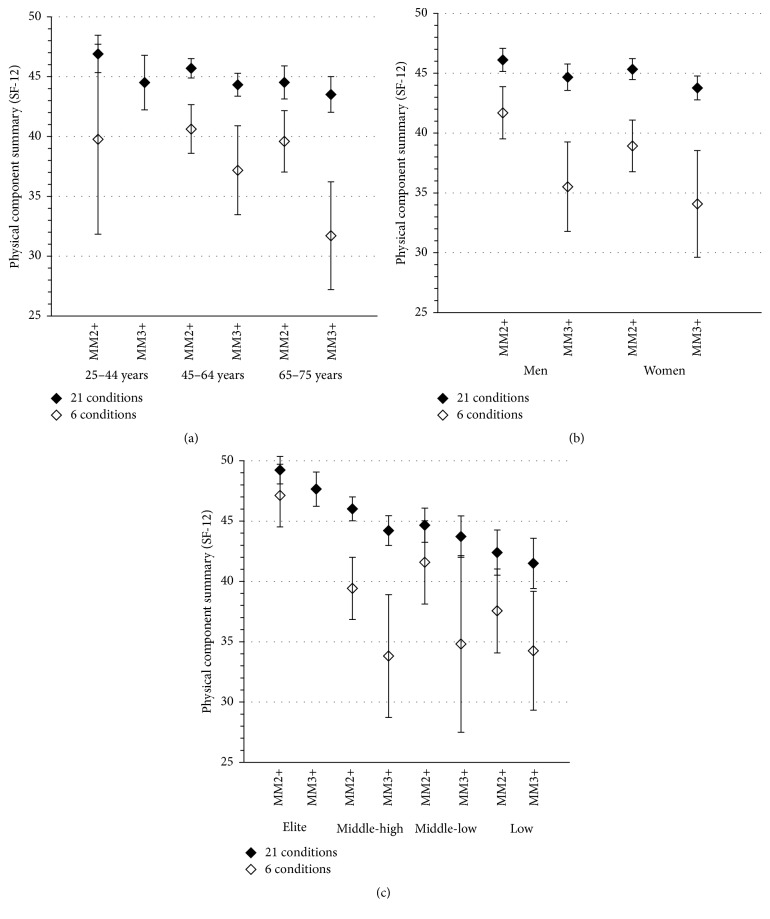
Estimates of the average level (mean values and 95% confidence intervals) of the physical component of health-related quality of life in individuals with multimorbidity, depending on the multimorbidity measure, stratified by age (a), gender (b), and socioeconomic status (c). Operational definitions of multimorbidity: MM2+: having two or more chronic conditions; MM3+: having three or more chronic conditions. Estimates were not computed in the case of multimorbidity measured as MM3+ based on the 6-condition list for young people “25–44 years” and for the “elite” group, due to insufficient subsample size (4 and 2 individuals, resp.).

Analyses stratified by age, gender, and socioeconomic level revealed similar patterns in the variations of estimates of quality of life within each of the subgroups of individuals successively considered (Figures 3(a), 3(b), and 3(c)). Using the 6-condition list consistently resulted in lower estimates of average physical health-related quality of life compared to using the 21-condition list. As in the main analysis, the variations observed in the stratified analyses were larger with the MM3+ operational definition than with the MM2+ operational definition.

Interestingly, using a reduced list of conditions to measure multimorbidity resulted in selecting people with substantially higher numbers of chronic conditions (Table 3). For example, the mean number of conditions as documented in the list of 21 conditions was 4.2 (SD 2.1) in individuals considered as multimorbid based on the MM2+ (21) definition while it was 6.1 (SD 2.3) in those considered as multimorbid based on the MM2+ (6) definition.

## 5. Discussion

This study suggests that different measures of multimorbidity result in significant variations in estimates of physical health-related quality of life within the same population. Our results show that using a reduced list of conditions leads to lower levels of estimated physical health-related quality of life in individuals with multimorbidity, independent of age, gender, and socioeconomic status, and whichever operational definition of multimorbidity was used (MM2+ or MM3+).

In fact, using a reduced list of conditions leads to the selection of a subgroup of individuals with an especially high number of existing chronic conditions, in comparison to the whole population considered as multimorbid based on a longer list. Each condition an individual has, whether documented or not, impacts his or her quality of life. It is therefore not surprising that people considered as multimorbid based on a reduced list of conditions have a higher number of existing chronic conditions while reporting lower physical quality of life.

The two lists used in this study were chosen for their contrast in terms of number of candidate conditions and because the six conditions of the reduced list were also included in the full list of 21 conditions. However, not one of these lists captures the whole range of the potential chronic conditions [36]. The use of any limited list, regardless of its length, necessarily implies a certain amount of unmeasured variability, due to unlisted conditions, and introduces a systematic bias towards the selection of individuals with higher degrees of multimorbidity. In that sense, using an open list of conditions to measure multimorbidity would result in a more accurate representation of reality, while being associated with other important challenges, such as reproducibility of the measure or optimal granularity in recording.

Beyond number, the nature of conditions considered in any list influences the estimated level of health-related quality of life. Among the 21 conditions from our full list, our 6-condition list included some which are among those with the highest impact on health-related quality of life (chronic obstructive pulmonary disease and stroke) and some which are among those with the lowest impact (hypertension or diabetes) [15, 16, 37]. We therefore believe that the variation observed in our study does not primarily result from the nature of conditions considered in the lists, but rather from the number of candidate conditions itself.

In our study, estimated prevalence of multimorbidity varied as much as 3.8% to 63.8% depending on the measure used. Moreover, the variations observed in the estimates of quality of life were 5.5 point units (means 45.7 to 40.2) with MM2+ and 9.4 points (means 44.2 to 34.8), with MM3+. These variations are larger than what is considered as the minimal clinically important difference for this score, namely, 5 points [38]. This illustrates how such methodological issues have the potential to considerably impact results and indicates that careful methodological considerations are required when measuring multimorbidity. The variations resulting from the alternative use of any other multimorbidity measures might be of different intensity from those observed in our study, but more stringent measures of multimorbidity will necessarily tend to identify smaller and sicker subgroups.

In addition to the quality of life, many other outcomes have been associated with the number of chronic conditions, such as disability [10, 13], psychological distress [12, 39], mortality [40], healthcare utilization [12, 41], or costs [41]. We believe that the choice of multimorbidity measure, and especially the length of the list of conditions, may also induce substantial variations when estimating the outcomes in multimorbid patients. Although stringent measures may be relevant for clinical purposes, short lists of conditions should be avoided in epidemiological studies: the shorter the list, the more biased the estimates of multimorbidity prevalence and related outcomes.

This study was based on data from the PRECISE cohort that constituted a representative sample of the Quebec general population at baseline [25]. The sample included in this study underrepresented young and deprived individuals, who were not as many to return their questionnaire (data not shown). However, the aim of this study was not to provide estimates of the physical component of health-related quality of life to be extrapolated to the general population, but rather to document variation in estimates resulting from using different multimorbidity measures. It is unlikely that this response bias has contributed to the results in any way. Prevalence of certain chronic conditions and of multimorbidity, as well as health-related quality of life, has been shown to present substantial international variations [10, 42, 43]. However, the impact of chronic conditions on health-related quality of life seems to be quite similar across countries [44]. Therefore, although our estimates may be not generalized to other populations, some variation in health-related quality of life could also be observed, within other cultural environments, when using different multimorbidity measures. We had to rely on the self-reported presence of chronic conditions to measure multimorbidity and, hence, either overreporting or underreporting may have occurred. This might affect the prevalence of some conditions in the sample. However, we do not think that this possibility would have an important impact on the differences observed with the use of different lists of conditions, which is the main message of this study.

## 6. Conclusion

Previous research had hypothesized that heterogeneity in multimorbidity measures may generate variability when studying quality of life in multimorbid individuals. This study demonstrated how different multimorbidity measures actually result in significant variation in the estimates of physical health-related quality of life within the same population. It argues for careful methodological consideration when measuring multimorbidity and its association with different outcomes. Standardization of the measure of multimorbidity is needed to allow the comparison of the results across different studies on multimorbidity.

In this regard, we recommend the use of a list of candidate conditions that is sufficiently long. Determining the ideal length is beyond the scope of this study, but it should be a compromise between lists that are too short, which will produce seriously biased estimates (6-condition lists being in this category) and lists that are too long, which can be difficult to manage. In order to reach a satisfying compromise, we suggest that both prevalence and impact for individuals and communities should be taken into account when choosing which conditions to include in the list.

## Figures and Tables

**Table 1:** Sociodemographic characteristics of the study population (*N* = 1,710).

	Study population
Age, mean (SD) *22 missing*	51.3 (12.5)
Males, *n* (%):	693 (40.5)
Annual household income (CAN$), *n* (%) *46 missing*	
Less than 20,000	196 (11.5)
20,000 to 49,999	699 (40.9)
50,000 or more	769 (45.0)
Education level, *n* (%) *8 missing*	
Less than high school	376 (22.0)
Completed high school	521 (30.5)
College/university	805 (47.1)
Socioeconomic status^a^, *n* (%) *66 missing*	
Low	230 (13.5)
Middle-low	345 (20.2)
Middle-high	733 (42.9)
Elite group	336 (19.6)

SD: standard deviation; ^a^Socioeconomic classes were derived from a data-driven combination of the following variables: education level, perceived financial situation, house ownership, presence or absence of medical insurance, and possession of a retirement plan.

**Table 2:** Prevalence of each individual chronic condition in the study population (*N* = 1,710).

	*n* (%)
**Angina/coronary artery disease **	124 (7.3)
**Asthma **	176 (10.3)
**Chronic obstructive pulmonary disease **	66 (3.9)
**Diabetes **	145 (8.5)
**Hypertension **	477 (27.9)
**Stroke **	21 (1.2)
Back pain	353 (20.6)
Osteoarthritis	361 (21.1)
Rheumatoid arthritis	44 (2.6)
Osteoporosis	94 (5.5)
Other illnesses of joints or limbs, lasting for 6 months or more	205 (12.0)
Cancer (within the past 5 years)	65 (3.8)
Cholesterol, elevated	439 (25.7)
Colon problem	123 (7.2)
Congestive heart failure	37 (2.2)
Depression	221 (12.9)
Hard of hearing	213 (12.5)
Overweight	1000 (58.5)
Stomach problem	356 (20.8)
Thyroid disorder	198 (11.6)
Vision problem	137 (8.0)

Conditions in bold are common to the two lists: full list (21 conditions) and reduced list (6 conditions).

**Table 3:** Main characteristics of the individuals with multimorbidity, depending on the multimorbidity measure.

Measure of multimorbidity	MM2+		MM3+	
	21 conditions (*n* = 1091)	6 conditions (*n* = 237)	21 conditions (*n* = 836)	6 conditions (*n* = 66)
Age: mean (SD)	55.0 (11.4)	59.0 (9.7)	56.3 (10.6)	59.5 (8.7)
Males: *n* (%)	455 (41.7)	111 (46.8)	352 (42.1)	33 (50.0)
Socioeconomic status^a^: *n* (%)				
Low	165 (15.1)	50 (22.4)	137 (16.4)	17 (28.3)
Middle-low	227 (20.8)	47 (21.1)	176 (21.1)	13 (21.7)
Middle-high	456 (41.8)	96 (43.0)	346 (41.4)	28 (46.7)
Elite group	200 (18.3)	30 (13.5)	143 (17.1)	2 (3.3)
Chronic conditions: mean number (SD)				
As documented by the 21-c. list^b^	4.2 (2.1)	6.1 (2.3)	4.8 (1.2)	7.8 (2.4)
As documented by the 6-c. list^c^	0.9 (0.9)	2.3 (0.6)	1.1 (0.9)	3.2 (0.5)

SD: standard deviation; ^a^Socioeconomic classes were derived from a data-driven combination of the following variables: education level, perceived financial situation, house ownership, presence or absence of medical insurance, and possession of a retirement plan; ^b^21-condition list; ^c^6-condition list.

Missing data for each variable are not reported in this table because their number differed depending on the group considered. They ranged from 2 missing values (age, MM3+, 6 conditions) to 43 missing values (socioeconomic status, MM2+, 21 conditions).
